# Investigating the Relationship Between Overexposure to Ultraviolet Radiation and Rheumatoid Arthritis Using National Health and Nutrition Examination Survey (NHANES) 2015–2016 Data

**DOI:** 10.7759/cureus.28298

**Published:** 2022-08-23

**Authors:** Rukevwe Madusor, Ahmed Bedaiwi, Koko Womas, Wanying Pei

**Affiliations:** 1 Epidemiology, University of Nebraska Medical Center, Nebraska, USA

**Keywords:** " "arthritis, sunburn, vitamin-d, autoimmune disorder, rhuematoid arthritis

## Abstract

Background

Research on the association between sunburn and autoimmune diseases including rheumatoid arthritis is scarce. To date, no study has looked at the relationship between over-exposure to ultraviolet (UV) radiation indicated by sunburn and rheumatoid arthritis (RA). We addressed this gap using the United States National Health and Nutrition Examination Survey (NHANES) database following a hypothesis that no relationship exists between sunburn and rheumatoid arthritis.

Methods

A cross-sectional study was performed using the United States NHANES data cycle from 2015 to 2016. Participants without rheumatoid arthritis and sunburn data have been excluded from this study. Chi-square test and survey-weighted logistic regression were conducted to study the strength of the association between overexposure to UV radiation indicated by sunburn and RA. Some RA risk factors have been included in the study to identify effect modifiers and confounders for building the parsimonious model.

Results

Based on the odds ratio (OR), individual overexposure to ultraviolet radiation had no higher or lower chance of reporting a diagnosis of RA [OR=0.87, 95% confidence interval (CI): 0.46 - 1.64]. Age was identified as a confounder. The Adjusted Odds Ratio (AOR) when accounting for age was AOR=1.09, 95% CI: 0.59 - 2.03. In the final model, there was not enough statistical evidence to conclude an association between sunburn and RA after adjusting for age.

Conclusions

Using the NHANES data to analyze the relationship between overexposure to UV radiation indicated by sunburn and RA; the analyses results suggested that sunburn may not be associated with higher or lower odds of developing rheumatoid arthritis.

## Introduction

Rheumatoid arthritis (RA) is an autoimmune disease that accounts for one of the most common chronic inflammatory arthritis. Severe cases of RA can result in disability, socioeconomic cost, and early death [1,2]. The disease primarily affects the joint causing joint pains, swelling, and damage throughout the body [3,4]. Although the etiology of RA is not completely understood, the pathogenesis of the disease is multifactorial and has been linked to autoantibodies and immune complexes [5]. Genetic, environmental, and lifestyle factors have also been implicated in the development of RA [6]. Following the discovery of vitamin D receptors in the cells of the immune system, vitamin D has been reported to have immunoregulatory properties. These properties have been demonstrated in studies indicating the role of vitamins in the immune system [7,8]. Ultraviolet radiation (UV) is important in the body’s production of vitamin D and low serum vitamin D might be related among other factors to reduced sunlight exposure [9].

Despite the beneficial immunomodulation reported by ultraviolet radiation-induced vitamin D synthesis, several available studies have shown conflicting results [10]. Some studies have reported a protective relationship between UV and some autoimmune diseases such as RA, multiple sclerosis, and insulin-dependent diabetes [11]. Other studies have shown that UV rays increase the risk of systemic lupus erythematosus and dermatomyositis [12,13]. No study however has examined the relationship between overexposure to UV radiation, as measured by sunburn, and RA. The purpose of this study is to determine if there is a relationship between sunburn and a history of rheumatoid arthritis.

## Materials and methods

Data source and study population

The data source for this study was collected from the National Health and Nutrition Examination Survey (NHANES) 2015 to 2016 cycle. NHANES is a national mobile health survey designed to assess adults' and children’s health and nutritional status in the United States. NHANES data utilized a complex, multistage, and stratified design with oversampling. NHANES is a representative sample of the non-institutionalized U.S. population. The NHANES protocols were approved by the National Center for Health Statistics Institutional Review Board and consent had been obtained from all participants [14].

**Figure 1:**
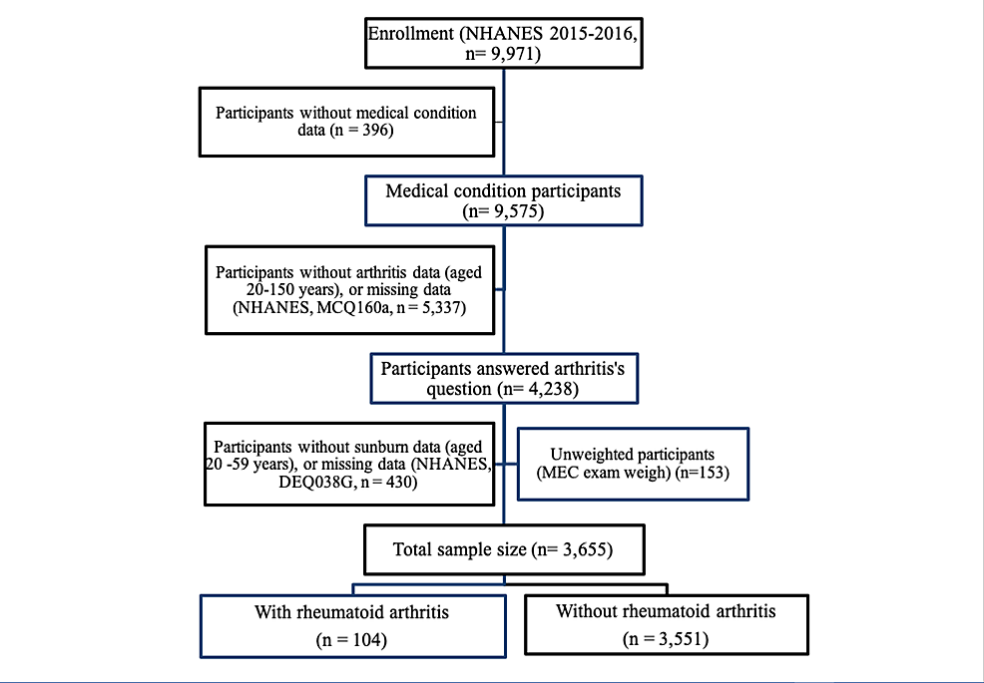
A Flow Diagram of the Participant’s Selection Criteria.

The study population in NHANES from the 2015 to 2016 cycle was 9,971 participants of all ages. Participants 20 years or older were asked the following questions regarding arthritis: “Has a doctor or other health professional ever told you that you had arthritis?” The second question was “Which type of arthritis was it?” Then, only participants between 20 to 59 years of age were asked if they had sunburn in the past year. Hence, the selection criteria for this analysis were participants in the NHANES 2015 to 2016 circle between 20 and 59 years of age with the complete outcome and exposure data of interest (Figure 1). Skip patterns were identified for participants who answered no to the arthritis question.

Study variables

Rheumatoid Arthritis and Sunburn Exposure

The outcome variable was rheumatoid arthritis (RA) and diagnosis of rheumatoid arthritis was self-reported and coded in medical condition data in NHANES data. The follow-up question once a participant was identified to have RA was: “Which type of arthritis was it?” Therefore, participants were located in cases and control groups based on the type of arthritis disease they had. The exposure variable was sunburn and sunburn exposure was identified from the dermatology data in NHANES and was based on self-reported data. Participants were asked, “How many times in the past year have you had a sunburn?” In this study, the variables included patients with or without sunburn exposure at least once a year.

Demographic Data

The study variables included age, gender, race/ethnicity, marital status, educational level, and the ratio of family income to poverty, which were collected and recorded in the NHANES database. Age was coded in years from 0 to 79, and participants that were 80 years or older were coded as 80. Race/ethnicity was reported as Mexican American, Hispanic, Non-Hispanic White, Non-Hispanic Black, and other races. In this study, Non-Hispanic White and Black were in one category, and Hispanic and other races (Mexican American, Hispanic, and other races) were in another category. Marital status was also self-reported and coded as married or not married. Educational level was self-reported and recorded as participants who completed up to 11th grade with no diploma or high school and above. The ratio of family income to poverty was self-reported from 0 to five based on the U.S. Department of Health & Human Services classification [15]. In this analysis, the ratio of family income to poverty was categorized into < 2.00 and ≥ 2.00. Most of the analysis coding was modified to have a sample size of at least 30 participants in each category.

Examination Data

Body Mass Index (BMI) was extracted from NHANES examination data. In NHANES data, BMI was a continuous variable and calculated based on the height and weight of participants (kg/m2), ranging from 11.5 to 67.3. Based on the Centers for Disease Control and Prevention (CDC), BMI was categorized into two groups: not obese participants whose BMI < 30 (including underweight, normal, and overweight), and obese participants whose BMI ≥ 30 (including obese, and above) [16].

Questionnaire Data

Other variables such as smoking alcohol and physical activity had been chosen from NHANES questionnaire data to study any effect modifier or confounder based on the literature. Smoking status was self-reported. Two questions had been used to categorize the variable: “Have you smoked at least 100 cigarettes in your entire life?”, and “Do you now smoke cigarettes?” Cigarette use was grouped into three categories. Never smokers, who had smoked less than 100 cigarettes in their life. Former smokers who had smoked at least 100 cigarettes in their life but quit smoking. Lastly, current smokers who had smoked at least 100 cigarettes in their life, and were still smoking [17].

Alcohol was also self-reported. Participants were asked, “In any one year, have you had at least 12 drinks of any type of alcoholic beverage? A drink is indicated by 12-oz. beer, a 5 oz glass of wine, or one and a half ounces of liquor." Alcohol was categorized into two groups: participants who drink ≥ 12 drinks or ˂ 12 drinks per year.

Regarding physical activity, participants were asked to report minutes from 0 to 1380 of sedentary activity. The following question was used “Do not include time spent sleeping, how much time do you usually spend sitting on a typical day?" In the analysis, sedentary activity was converted to hours and grouped into two categories: from 0 ˂ seven hours, and from seven to 23 hours.

Statistical Analysis

The analysis included the following variables: masked variance pseudo-stratum (SDMVSTRA), masked variance pseudo-cluster (SDMVPSU), and full sample two-year MEC exam weight (WTMEC2YR). These variables were included to estimate the real statistical results if the entire sampling frame had been surveyed. All data were merged into one data set by a sequence number (SEQN). Age was a continuous variable and was summarized using mean and standard deviation (SD). Categorical variables were summarized as numbers and proportions (%) of each category in a variable. Exact and non-exact duplicates were checked. Missing data were excluded from the analysis and reported. Binary logit regression was used to determine the relationship between parameters and RA. Therefore, crude and adjusted odds ratios and the Breslow-Day test had been looked at to decide which parameters are confounders or effect modifiers. The analysis was conducted with SAS statistical software (version 9.4, SAS Inc., Cary, NC, USA).

## Results

Study sample

The NHANES cycle from 2015-2016 had 9,971 subjects in total; however, some questions in the NHANES questionnaires were not applicable for certain ages. The arthritis question in the NHANES questionnaire was asked to 9,755 subjects, only 4,238 of whom answered the question. In terms of sunburn data, the dermatology data only had 3,808 participants (aged 20-59). Furthermore, unweighted participants had been removed from the total sample size; therefore, another 153 subjects had been excluded from the study. The total sample size for those with RA and without RA was 104 and 3,551, respectively (Figure 1).

The mean age of the participants with RA (46.65 ± 2.73) was lower than those who did not have RA (39.29 ± 0.52). Furthermore, females with RA had a higher proportion than males by 63.64 and 36.36, respectively. Most of the participants without RA had at least a high school degree or above. RA patients seemed to have higher BMI and to drink at least 12 drinks or more. Smoking status and sedentary hours were not affecting RA patients. Lastly, race/ethnicity, marital status, and the ratio of family income to poverty were approximately the same proportion for participants with and without RA. Some participants had missing data in certain variables. These missing data were not included in the analysis and have been reported (Table 1).

**Table 1:** Characteristics of Adults (aged 20-59) by Rheumatoid Arthritis, NHANES 2015-2016 (N=3,808)

	Rheumatoid Arthritis (RA)								
	Yes (n=104)^ a ^			No (n =3,551)					
Participants Characteristics	No.	n (millions, %) e	No.		n (millions, %) e		OR (95% Cl)^ b^		
Sunburn									
Yes	41	1.7 (43.83)	1,244		78.0 (47.32)		0.87 (0.46 – 1.64)		
No	63	2.2 (56.17)	2,307		86.7 (52.68)		1.00		
Age, years^ f^	46.65 ± 2.73	3.9	39.29 ± 0.52		164.7		1.06 (1.04 – 1.09)		
Sex									
Male	38	1.4 (37.34)	1,686		81.5 (49.45)		0.61 (0.33 – 1.13)		
Female	66	2.5 (62.66)	1,865		83.2 (50.55)		1.00		
Race/Ethnicity^ c^									
Hispanic/Others^ d ^	51	1.2 (31.88)	1,707		45.8 (27.83)		1.21 (0.71 – 2.08)		
Non-Hispanic white/Black	53	2.7 (66.12)	1,844		118.9 (72.17)		1.00		
Education^ c^									
< High School	30	0.8 (21.56)	716		22.7 (13.79)		1.72 (1.06 – 2.80)		
High school graduate/college or higher	74	3.1 (78.44)	2,835		142.0 (86.21)		1.00		
Marital Status^ c^									
Married	55	2.2 (55.70)	1,766		86.1 (52.26)		1.00		
Not married	49	1.7 (44.30)	1,784		78.6 (47.74)		0.87 (0.53 – 1.43)		
Missing	0	-	1		-		-		
Body Mass Index (kg/m^2^)^ c^									
Not obese	45	1.6 (40.41)	2,116		99.7 (61.18)		1.00		
Obese	59	2.3 (59.59)	1,405		63.3 (38.82)		2.32 (1.29 – 4.19)		
Missing	6	-	177		-		-		

	Rheumatoid Arthritis (RA)				
	Yes (n=104)^ a^		No (n =3,551)		
Participants Characteristics	No.	n (millions, %) e	No.	n (millions, %) e	OR (95% Cl)^ b^
Ratio of family income to poverty ^c^					
˂ 2.00	47	1.5 (41.62)	1,532	53.0 (34.52)	1.35 (0.87 – 2.11)
≥ 2.00	46	2.2 (58.38)	1,707	100.5 (65.48)	1.00
Missing	11	-	318	-	-
Alcoholic drinks^ c^					
<12 drinks/year	34	1.2 (32.01)	888	32.4 (21.72)	1.70 (0.85 – 3.40)
≥12 drinks/year	60	2.5 (67.99)	2,248	116.7 (78.28)	1.00
Missing	10	-	415	-	-
Smoking^ c^					
Never smoker	53	1.9 (48.89)	2,223	98.3 (59.73)	1.00
Current smoker	32	1.0 (24.99)	738	33.9 (20.58)	1.48 (0.85 – 2.59)
Former smoker	18	1.0 (26.12)	588	32.4 (19.69)	1.62 (0.91 – 2.89)
Missing	1	-	2	-	-
Sedentary hours					
0 to < 7 hours	68	2.4 (61.36)	2,150	93.1 (56.88)	1.00
7 to 23 hours	34	1.5 (38.64)	1,375	70.6 (43.12)	0.83 (0.39 – 1.77)
Missing	2	-	26	-	-

Crude odds ratios (OR) for RA patients had been calculated for each participant’s characteristics to determine the association with the covariates. The odds ratio of having RA for people who were overexposed to UV indicated by sunburn is 0.87 compared with those who did not have sunburn exposure (95% Cl: 0.46 - 1.64). The crude odds ratio between RA and sunburn did not show an association. Some associations had been found for some covariates, such as age, education, and BMI. The odds ratio of having rheumatoid arthritis disease is estimated to be increased by a factor of 1.06 for every one-year increase in age (95% Cl: 1.04 - 1.09). Regarding education, the odds ratio of having rheumatoid arthritis for people whose education status is less than high school is 1.72 times higher compared to those with at least a high school degree (95% Cl: 1.06 - 2.80). Lastly, the odds ratio of having rheumatoid arthritis for obese (BMI ≥ 30) is 2.32 times higher compared to not obese people (95% Cl: 1.29 - 4.19). The odds ratios for other participants' characteristics did not show an association with RA (Table1).

Breslow-day test within 0.1 significance level was used for each predictor to identify effect modifiers. If the Breslow-day was not statistically significant, meaning more than a 10% difference from the crude, odds ratio and adjusted odds ratio for each predictor were calculated to identify the confounders. No effect modifiers were found. Age was the only confounder (OR=1.06). After adjusting for age, the adjusted odds ratio (AOR) was measured to build the model. Age was treated as a continuous variable after testing the linearity of the logit. The linearity of the logit for age was linear. Finally, in the final model after adjusting for age, the odds ratio among RA patients who have sunburn at least once a year is 1.09 compared to those without RA (95% Cl: 0.59 - 2.03). In this snapshot study, an association could not be determined between sunburn and having RA.

## Discussion

Research on the association between sunburn and autoimmune diseases including rheumatoid arthritis is scarce. The purpose of this study was to analyze the relationship between sunburn and rheumatoid arthritis. In this cross-sectional study, as a result of the logistic regression analysis, individuals with sunburn had no greater odds of reporting having rheumatoid arthritis than those who do not have sunburn. (OR=0.87, 95% CI =0.47-1.60). There was no association between sunburn and rheumatoid arthritis. Our finding is congruous with the cohort study by Arkema, who found no association between ultraviolet radiation and rheumatoid arthritis among women in the Nurses’ Health Study II (NHSII) cohort [18]. Our study was however not in agreement with some previous work by Ponsonby who reported that ultraviolet radiation was protective of rheumatoid arthritis and Arkema who found that exposure to UV radiation was associated with a lower rheumatoid arthritis risk in an NHS cohort, but not NHSII [10,18]. This disparity may be because of a weakened association due to the small sample size in our study. Results from the Iowa women's health cohort study suggested that greater intake of vitamin D which is produced by ultraviolet radiation may be associated with a lower risk of rheumatoid arthritis in older women [19]. Reports from this study are also not in support of findings from our study. This discrepancy may be attributed to differences in demographics such as age and race, as participation of mainly elderly white women between the ages of 55-69 years were included in the Iowa women’s health cohort study. Some possible mechanisms have been suggested for the protective role offered by UV radiation such as a UVR-induced increase in serum vitamin D levels and vitamin D has been shown to have beneficial effects as an immunosuppressant [10,19]. Although the result of studies has suggested a possible role of UV radiation in reducing the risk of rheumatoid arthritis, result from our study suggest overexposure to UV radiation indicated by sunburn may not be associated with rheumatoid arthritis.

This study is significant in that it is the first study to analyze the relationship between sunburn and rheumatoid arthritis and this would serve as a landmark for other studies which may establish the biological link between sunburn and rheumatoid arthritis and thus, predict or prevent rheumatoid arthritis through prevention and early detection of sunburn. The strength of this study is that questions on arthritis and sunburn from NHANES were used and we believe the validity of the questionnaires is reasonable. There are some limitations to this study. This study is a cross-sectional study, in light of this, the cause and effect relationship cannot be determined in this study. The sample size was a critical problem in our study and the result may be different if we had a large enough sample size and analyze the numbers of sunburns associated with rheumatoid arthritis.

## Conclusions

There is no relationship between sunburn and rheumatoid arthritis. The result from this study suggests sunburn may not be associated with a higher or lower odds of developing rheumatoid arthritis. Further studies may be needed to address the limitations of this study as well as to corroborate this finding.
